# Place attachment among rural migrants and returnees: case of Shuangfeng County, China

**DOI:** 10.3389/fpsyg.2023.1279679

**Published:** 2023-11-28

**Authors:** Lei He, Yingming Mao, Takeshi Kinoshita

**Affiliations:** Graduate School of Horticulture, Chiba University, Chiba, Japan

**Keywords:** native and destination place, rural area, migrants and returnees, place attachment, rootedness, rural sustainability, urbanization, typology

## Abstract

In the mobile era, place attachment among rural migrants and returnees has become dynamic and diversified. However, research on place attachment to native place among rural migrants and returnees is limited. The focus of previous research has primarily been on the destination place attachment of rural migrants, which makes it difficult to gain a comprehensive understanding of the place attachment among both rural migrants and returnees. This study aims to investigate the state of place attachment to both native and destination places among rural migrants and returnees originating from the same birthplace. It explores their place attachment after migrating from rural areas to cities. A quantitative research approach was adopted, garnering questionnaire responses from 274 rural migrants and returnees, all born in Shuangfeng County, Hunan Province. The questionnaire encompassed a Likert scale for measuring place attachment, as well as sociodemographic statistical information. Exploratory factor analysis and confirmatory factor analysis were conducted to ascertain the reliability and validity of the questionnaire. Based on the factor scores of place attachment to both places from migrants and returnees, a two-step cluster analysis identified three types of migrants and two types of returnees. Chi-square tests revealed significant differences among migrants in terms of property ownership, educational level, marital status, presence of children, age at departure, and time away from hometown. The study discovered that, regardless of being a migrant or returnee, the overall attachment to hometown was stronger than that to the current or previously inhabited city. In the context of existing literature primarily concerned with the integration of rural migrants into urban areas, this paper offers a fresh research perspective, highlighting the significance of emotional ties to one’s hometown for rural migrants. The findings of this paper provide direction and a theoretical basis for rural areas to attract return migration and for urban regions to facilitate the integration of migrants.

## 1 Introduction

### 1.1 Background

In the process of urbanization, the mass migration of populations from rural areas has emerged as a global issue (Mendola, 2012). In China, this phenomenon can be traced back to the 1970s and has persisted to the present day (Goodman, 2014). The large-scale population mobility in China is underpinned by the economic Reforms and Opening-up policies initiated in 1978, which have increasingly allowed labor force movements to be dependent by market instead of centrally planned economy. In addition, policies that previously restricted population flows have been progressively abolished, such as the dissolution of the people’s communes in 1983, the issuance of the national ID card in 1986, and the termination of the food rationing system in 1988, laying the groundwork for labor transfer (Li et al., 2014). The focus on economic development, combined with substantial urban–rural disparities, has driven waves of migration, predominantly comprising rural migrants moving toward coastal cities where opportunities are concentrated. According to the UN’s WORLD URBANIZATION PROSPECTS 2018 report, since 2009, China has had fewer rural residents than urban residents, a trend that is predicted to continue until 2050.

The influx of migrants from rural areas to cities has significantly contributed to China’s tremendous economic growth. However, for the rural migrants themselves, migration means leaving their familiar surroundings and entering a strange host city. Concurrently, rural migrants find themselves in two distinctly different states of identity based on whether they hold local citizenship, known as “Hukou.” For the vast majority of rural migrants without “Hukou,” they are excluded from urban citizenry benefits. This exclusion impacts their housing options, their children’s education, and their future pension entitlements, creating disparities between them, the local urban residents, and rural immigrants who have acquired urban “Hukou” (Li and Song, 2009). Regardless of the duration of their stay, they may still be considered “outsiders” (Du et al., 2018). Fan (2002) articulated the enduring nature of the immigrant and the transitory condition of the migrant with the terms “elite” and “outsider” (Fan, 2002).

As population mobility increases, the relationship between migrants and their local environments has become a focal point for environmental psychologists and human geographers (Lewicka, 2011b). For this study, “rural migrants” are defined as individuals who were born in rural areas and left their place of birth at a later stage in life, including immigrants who have acquired urban “Hukou” and transitory rural migrants. “Returnees,” on the other hand, refer to those who were born in rural areas, have experienced life in the city, and subsequently chose to return to and reside in the rural areas.

### 1.2 Background to the study

As research on the relationship between people and places deepens, a wealth of empirical studies from the West have attested to the universality of place attachment and its compatibility with mobility. The traditional view of place attachment suggests that an individual’s attachment is directed toward a single location and that migration away from this place diminishes the attachment. This perspective has been challenged as the study of place attachment has evolved (Gustafson, 2001). Subsequent empirical studies have demonstrated that attachment is not confined to a single locale but is linked to multiple networks of interests and practice communities (Jensen, 2009). Rural migrants, despite leaving their home communities and residing in distant locales, still maintaining strong attachments to their hometown. Their attachment is sustained through connections with their home communities and does not necessitate living within those rural communities (Barcus and Brunn, 2010). In other words, although economic development and urbanization have transformed the nature of place attachment, they have not eradicated it. Instead, an active form of attachment has supplanted the traditional form (Lewicka, 2011b). However, within the unique urban–rural context of China, the impact of migration on place attachment has yet to be explored.

Over the past several decades, China has witnessed a significant increase in mobility and the movement of internal migrants. Consequently, numerous studies have emerged on the subject of internal migration in China (Gao and Wang, 2022; Xie et al., 2022; Zhan et al., 2022). Previous research has primarily focused on the difficulties that rural migrants faced in integrating into new urban area and their satisfaction with life. These studies have indicated that community-based prejudice leads to a low satisfaction with the community among rural migrants (Du and Li, 2010). Despite residing in cities, the difficulty in obtaining urban “Hukou” (Chan, 2010) has resulted in a disparity in social welfare between rural migrants and urban locals, with non-resident migrants being marginalized (Keung Wong et al., 2007; Démurger et al., 2009). Institutional discrimination (Chen, 2013) hinders the rural migrants’ identification with the city, creating a negative perception of urban life (Du, 2018). Previous research lacks a comprehensive understanding of the attachment that rural migrants and returnees have toward their hometowns and destinations. Therefore, it is meaningful to study the attachment of migrant workers and returnees from the same birthplace to their place of origin and destination from the perspective of their native place.

### 1.3 Research aim and article overview

However, we have noticed that the attachment of rural migrants to their native place has not been thoroughly investigated, and attachment to the destination place constitutes only a part of rural migrants’ personal life narratives. Conducting surveys on urban attachment among rural migrants solely at the destination is insufficient for capturing the entirety of their attachment experiences and does not allow us to further understand the compatibility and conflict between migration and attachment. In this study, we will investigate the attachment of rural migrants from the same rural area (including relocated rural migrants and returnees who have experienced migration) to both their native and destination places in order to fill this research gap. The aim of this research is to enhance our understanding of place attachment among rural migrants and returnees, which will, in turn, contribute to a deeper comprehension of the relationships between multiple places, and place attachment. This paper poses the following research questions: Is there a difference in place attachment between migrants and returnees? Do rural migrants and returnees possess a certain type of place attachment as a distinctive group characteristic? Are there differences in social demographic data between groups with different place attachments?

Following this, we will review the literature related to place attachment, as well as the global rural migration and returnees. In the method section, the technical routes of this article, along with the sampling methods, sample characteristics, and analytical tools used for quantitative analysis will be presented. In the results section, we will introduce the place attachment of rural migrants and returnees to their place of native and destination, as well as the three types of migrants and two types of returnees that have been clustered based on their attachment factor scores. Additionally, we will explore the differences in sociodemographic variables among these five groups. In the final part of the article, we discuss the results of our study, delve into its limitations, and provide suggestions for policy and management.

## 2 Literature

### 2.1 Dynamic place attachment

In this study, place attachment refers to the emotional bonds between an individual and the social and material environment of a place. Regarding the formation of place attachment, an individual’s attachment to a place is based on early life experiences and is reinforced by time spent at that specific location (Fullilove, 1996). Emotions connect all human experiences; thus, environments are imbued with meaning through the stable accumulation of human emotions (Tuan, 1977). The formation of place attachment is an interactive process, occurring on personal, collective, or cultural levels. It encompasses a wide array of locations and sentiments within a broader social context, characterized by dynamism and constant change (Manzo, 2003). As an individual’s life experiences expand, so does place attachment, dynamically evolving. Leaving an attachment site can disrupt place attachment due to severed social ties (Manzo et al., 2008). Newcomers to a place can develop an attachment to their current city of residence; if they also maintain relatively close ties with their native place, the attachment to that native place can be preserved (Bielewska and Jasku, 2017). Among all sociodemographic variables affecting changes in place attachment, including duration of residence, age, education, social status, home ownership, parenthood, and experience of migration (Lewicka, 2011b), the length of residence is one of the most frequently reported factors influencing changes in place attachment (Stedman, 2006), closely related to age (Shamai and Ilatov, 2005) and the experience of migration (Gustafson, 2002). Some studies assert that the contribution of migration experience to place attachment is much less than that of mere residence duration (Lewicka, 2011b), while others suggest migration can shift people’s attachment from a specific place to a new form of identity. In conclusion, the relationship between migration and place attachment requires further exploration.

### 2.2 Place attachment of rural migrants and returnees

Regarding the place attachment of rural migrants and returnees, previous research has mainly concentrated on their attachment to a single location, that is, only to cities or to hometowns, without comparative analysis of the differences in place attachment to native places and destination places. Studies focused on urban areas have often used place attachment as an indicator of migrants’ psychological integration (Wang and Fan, 2012) because it reveals the connections that rural migrants establish with the city. Rural migrants’ attachment to urban communities is positive, albeit lower than the overall population’s assessment (Du and Li, 2010). Homeownership influences their attachment to urban areas; rural migrants living in purchased apartments exhibit more attachment to the city than those residing in urban villages (Lin et al., 2021). Moreover, rural migrants’ neighborhood relations in urban communities receive the most attention in relation to their formation of attachment to the city (Du and Li, 2010; Wang et al., 2016; Wu et al., 2019; Lin et al., 2021). Xie, through a nationwide survey, found that the willingness to interact with locals has a positive effect on rural migrants’ urban attachment (Xie and Huang, 2022). For rural migrants, while they rely on the city’s functions for income, entertainment, and social needs, there is no correlation between the functional dimensions of the city and place attachment. Their attachment in the city is largely influenced by their attachment to their hometown (Qian and Zhu, 2014), including the land of their hometown (Zou et al., 2022). Furthermore, systemic discrimination experienced by rural migrants in the city, such as household registration, hinders the establishment of a positive place attachment to the city (Yang, 2013). Studies on returnees’ place attachment, and those from a native perspective, are significantly less than migrant studies from an urban perspective. Zhang (2013) explored the return process of rural female migrants, noting that although women actively participate in migration, returnee women’s lives in the countryside are still influenced by familial patriarchal power relations (Zhang, 2013). Sun et al. (2022) demonstrated a positive correlation between emotional attachment to one’s hometown and entrepreneurial success through a study of returnee entrepreneurs (Sun et al., 2022).

As stated above, the place attachment of migrants and returnees to their native place has not been thoroughly investigated. Current research on migrant place attachment emphasizes the perspective of the destination, which hinders a comprehensive understanding of the differences in place attachment of rural migrants and returnees to both places. Therefore, we argue that place attachment research from the perspective of the native place is necessary, and simultaneous investigation of place attachment to both the native place and destination place can effectively fill the aforementioned knowledge gap.

### 2.3 Measurement of place attachment

This article employs a quantitative approach to measure place attachment. Various instruments for measuring place attachment are available in the literature (Hernández et al., 2020). These range from single-dimensional measures to methods incorporating four or even five dimensions (Boley et al., 2021). Earlier studies did not measure emotions directly but instead used behavioral indicators such as participation in activities, neighborhood relationships, duration of residence, frequency of visits, and property status as proxies (Gerson et al., 1977; Riger and Lavrakas, 1981; Taylor et al., 1985; McAndrew, 1998). Consequently, the associated emotions were not assessed directly. Current measures of place attachment often employ single-dimensional, emotion-related assessment items. For example, these include pleasure (Jorgensen and Stedman, 2001), safety (Lewicka, 2008, 2011a) pride (Lewicka, 2008, 2011a), care (Feldman, 1996), responsibility (Shamai, 1991), and belonging (Feldman, 1996). Simultaneously, the applicability of theory and methodology is increasingly emphasized. Yeh and Yang (1997) developed a tripartite model of cognition, affect, and behavioral intention toward home (Yeh and Yang, 1997), which Du (2017) refined and validated in a survey of educated young migrants, proving the same method’s applicability to the larger urban context (Du, 2017). This included emotion measurement items such as affiliation, belonging, concern, responsibility, security, and pride. This paper has utilized the same contextualized attachment scale to measure the place attachment of migrants and returnees to their native place and destination place.

In this paper, we use quantitative research, and more sociologically meaningful findings are expected, considering that we are studying people as human beings. While quantitative research usually compares people across a range of dimensions, qualitative research usually ends with a typology, which is a more natural way of organizing the material obtained from interviews or observations. There is some literature on place attachment that attempts to combine qualitative and quantitative dimensions, and typology is the most appropriate approach they employ. Typological qualitative analysis and comparisons across various dimensions are increasingly prevalent in place attachment research. Hummon, based on qualitative interviews with urban residents, summarized five forms in which people may relate to their residential areas: two types of rootedness (everyday and ideological) and three types of sentiments (alienation, relativity, and placelessness) (Hummon, 1992). Building upon Hummon’s work, Lewicka conducted a large-scale, representative survey in Poland and identified two types of place attachment (traditional and active attachment) and three types of non-attachment (alienation, place relativity, and placelessness) (Lewicka, 2011a). Not only have attachment styles been typologized, but the individuals forming emotional bonds with places have also been classified. Jamieson interviewed 45 young people who either left or stayed in rural Britain, resulting in four attachment-based respondent types: attached migrants, detached migrants, attached stayers, and detached stayers (Jamieson, 2000). Engbersen surveyed Central and Eastern European laborers, deriving four migration patterns based on their attachment to destination and origin countries: circular migrants, binationals, footloose, and settlers (Engbersen et al., 2013). Du conducted a questionnaire survey among college students who grew up in Lake Chaohu and left their hometown as adults, identifying four types of migrants (Translocals, Departers, Aliens, and Settlers) and three types of returnees (the Trapped, the Bonded, and the Rooted) (Du, 2017). Despite these advancements, typological investigations of rural migrants and returnees based on attachment remain underexplored in the place attachment research literature.

Previous typological analyses have been based solely on the attachment to a fixed location. However, in today’s context where place attachment is becoming more dynamic and diversified, people’s form of attachment is no longer confined to a single specific place; attachments to multiple places, as well as both positive and negative attachments, have emerged in research. To address this research gap, we have employed a quantitative method to measure the place attachment of rural migrants and returnees to both their native place and destination place.

## 3 Materials and methods

**Figure 1:**
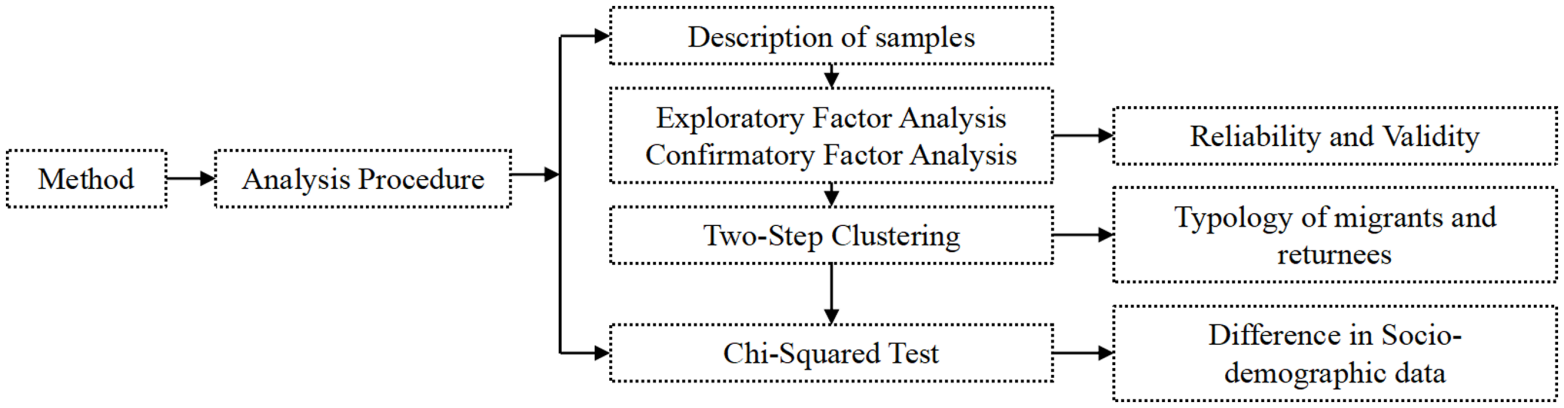
Technique routine.

Our survey site is in Shuangfeng County, Hunan Province, China, Shuangfeng County located in central region of Hunan province, which is a major province experiencing population outflow. From the year 2000–2020, the proportion of residents in rural areas of this county has declined to below 45%, making it a fitting location for our study. In order to investigate the place attachment of rural migrants and returnees originating from the same birthplace to their native and destination places, an online questionnaire method was chosen to conduct the survey with both migrants and returnees. In total, we garnered responses from 128 inter-provincial migrants, 100 intra-provincial migrants, and 59 returnees, amounting to 287 samples in all. Figure 1 shows technique routine of our method.

### 3.1 Study area

**Figure 2:**
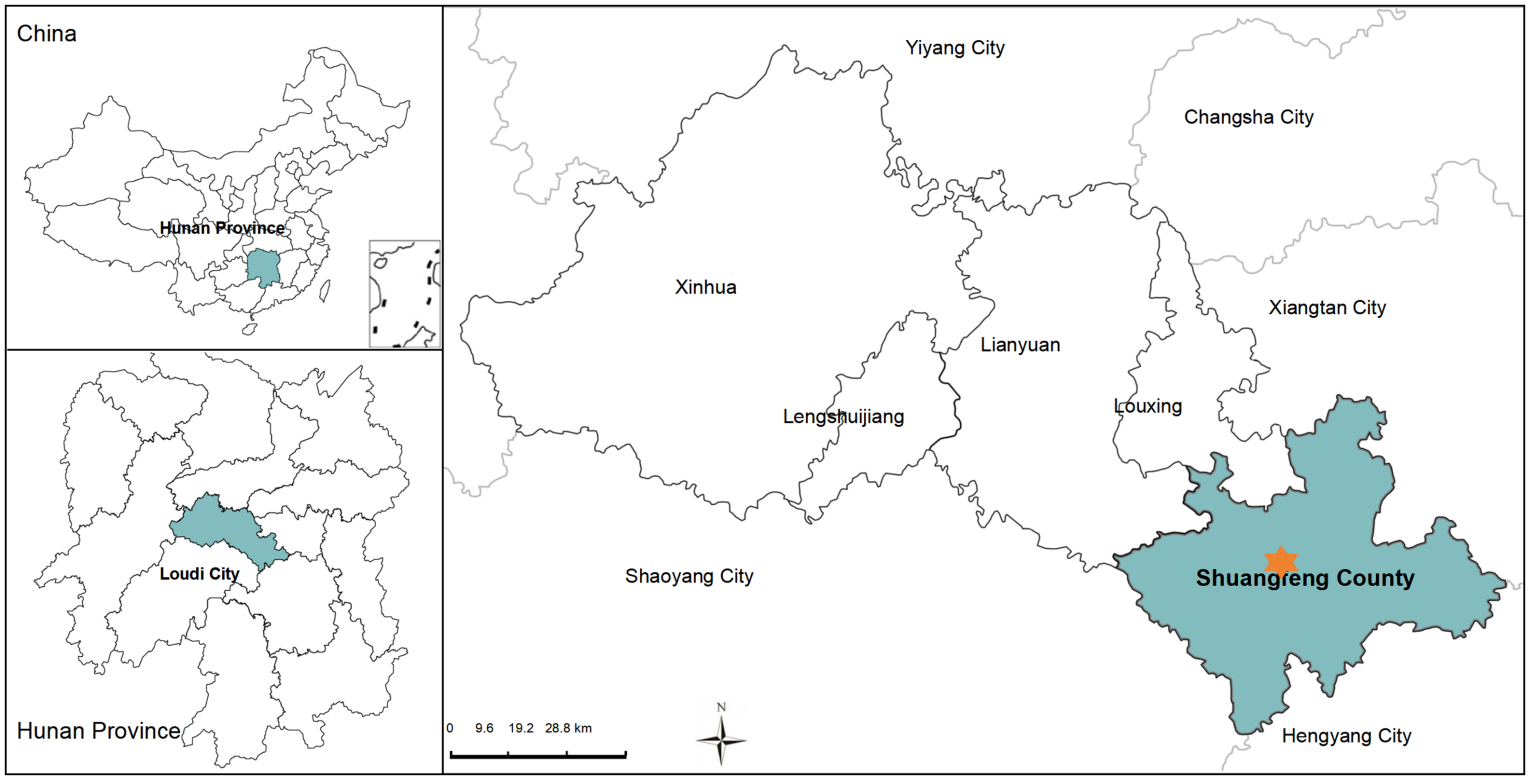
Study area.

Shuangfeng County (see Figure 2) is located in the central part of Hunan Province, China, covering a total area of 1,596 square kilometers. The population is approximately 680,000 (The People’s Government of Shuangfeng County, n.d.). As a county primarily focused on agricultural production, Shuangfeng County officially met the criteria for exiting China’s poverty list in 2019, ending its history as an “Impoverished County (People’s Government of Hunan Province, 2019).” The urbanization rate of Shuangfeng County is low, in the meantime the out-migration rate is high. Hunan Province, where the county is located, has the second-largest domestic floating population in China. Since the reform and opening of China, restrictions on population mobility have been relaxed, and leaving hometowns to work in more prosperous provinces has become a trend among Hunan residents (Qi et al., 2017). The rural resident population of Shuangfeng County has declined from 77% in 2001 to 41% in 2020 (The People’s Government of Shuangfeng County, 2021), reflecting the trend of rural depopulation and outflow, which makes it a representative area for studying rural out-migration. This demographic transition underlines the rationale for selecting Shuangfeng County as our study area. The primary destination for the floating population is Guangdong Province, located to the south. The vast majority of the migrating population consists of low-educated migrant workers. Path dependence, geographic proximity, and economic disparities are the main reasons for population migration (Cao et al., 2018).

### 3.2 Samples

From December 14–17th, 2019, a survey was conducted on rural migrants who were born and grew up in Shuangfeng. In China, the statistical method for floating population is to compare the registered permanent residence of the residents with the location of the town or street where they currently reside (National Bureau of Statistics of China, 2021a,b). If they are inconsistent and the time has been more than 6 months, they are classified as floating population. This statistical process occurs during the population census of the host city where the migrants currently reside. If a person born in a rural area moves to another place but does not change their registered permanent residence, they are still counted as a rural resident. It is quite common for the residence to be different from the registered permanent residence (National Bureau of Statistics of China, 2021a). When investigating the original place of population outflows, it is not possible to determine the proportion of migrants to the total population due to the lack of available sampling frames. Therefore, a non-probability sampling technique was used. An electronic questionnaire was used, which was distributed to participants through the snowball sampling method, with officials from the Shuangfeng County government invited to initiate the snowball process. The beginning of the electronic questionnaire introduced the researchers’ identity and contact information, and explained the purpose and process of the study to ensure participants were informed. To ensure the confidentiality and anonymity of the participants, no identifiable information was collected in the questionnaire. A total of 530 questionnaires was collected. After excluding questionnaires from respondents who were not born in Shuangfeng County and conducting integrity verification, a total of 287 valid questionnaires were obtained.

### 3.3 Variables

The survey items on attachment to the native place and destination place were extracted from a survey of student migrants in the Chaohu area of China conducted by Du (2017). Du drew on the experience of Yeh and Yang (1997) in their study of Chinese familism and proposed six items related to emotions: Affiliation, Belonging, Responsibility, Concern, Security, and Pride. As Jorgensen and Stedman argued, researchers need to pay attention to the applicability of theory and methods in different research contexts and contextualize them to better reflect the uniqueness of the context (Jorgensen and Stedman, 2001). The scale composed of these items also meets the call from Boley for a reduction in the diversity of scales used to measure attachment to place: researchers should adopt shorter, multi-item scales to reduce response burden and create more space for other constructs in the survey (Boley et al., 2021). In this study, each participant was required to answer 12 attachment-related questions. Each person needed to report their attachment to their native place, while migrants within the province and migrants from outside the province needed to report their attachment to their destination place. Returnees needed to report their attachment to the place they lived in when they migrated. The author used the terms “native place,” “destination place,” and “past residence” to inquire about the locations without limiting them to a specific scale (such as village, town, or county). These items were rated on a 5-point Likert scale, ranging from 1 (strongly disagree) to 5 (strongly agree).

In addition, the questionnaire asked about the participants’ migration experience and socio-demographic data. We inquired about the participants’ current status as intra-provincial migrants (migrants from rural areas to urban areas within the same province), inter-provincial migrants (migrants from rural areas to urban areas in another province), or returnees; the city where they currently reside (for intra-provincial or inter-provincial migrants) or the city where they used to work or study (for returnees); their age at the time of their first migration; the number of years they have lived, worked, or studied outside of their native place; whether they own property in the host city or in their native place. Along with these questions, we also asked about the participants’ age, gender, monthly income level, education level, and whether they have children. Previous research has shown that these factors are important in forming attachment to place, and by including these control variables, the study aims to explore the socio-demographic differences and contrasts between migrants and returnees.

### 3.4 Analytical procedures

The analytical process takes reference from Du’s survey of young urban migrants in Chaohu (Du, 2017). The first step in our analysis involves conducting a descriptive analysis of the scale results, wherein we calculate the overall mean values of attachment to both the native and destination places for the two groups: migrants and returnees. Additionally, we ascertain the scale mean values for three sub-groups—namely, intra-provincial migrants, inter-provincial migrants, and returnees—specifically in relation to their attachment to native and destination places. The second step encompasses performing exploratory and confirmatory factor analyses on the scale results, with the aim of validating the attachment scale. Subsequently, in the third step, we employ a two-step clustering method to generate sub-samples of three types of migrants (including intra-provincial and inter-provincial migrants) and two types of returnees from all the samples, categorizing them separately helps in more accurately studying the characteristics and experiences of these groups. By studying them separately, can delve deeper into the unique emotional experiences of each group. At last, we examine sociodemographic variables to investigate whether these types of migrants exhibit significant differences in their sociodemographic characteristics.

## 4 Results

### 4.1 Place attachment: descriptive results

Table 1 displays the responses of the participants to the 12 questions regarding attachment to the native place and destination place. Among the participants, 100 intra-provincial migrants and 128 inter-provincial migrants answered questions about attachment to their native place and their current place of residence. 59 returnees answered questions about attachment to their native place and their former place of residence, work, or study.

**Table 1:** Place attachments to native place and destination place (percentage).

	Affiliation	Belonging	Responsibility	Concern	Security	Pride
Native place (*n* = 287)						
Strongly disagree	2.8	1.4	3.1	2.1	2.8	2.8
Disagree	4.9	13.2	11.1	0.7	9.4	10.8
Neutral	6.6	9.1	8.4	8.4	9.8	9.4
Agree	62.0	60.3	65.2	69.7	62.0	63.4
Strongly agree	23.7	16.0	12.2	19.2	16.0	13.6
Mean	3.99	3.76	3.72	4.03	3.79	3.74
Destination place (*n* = 287)						
Strongly disagree	2.4	3.1	2.4	0.3	2.4	1.7
Disagree	12.5	25.4	11.5	5.6	9.4	9.1
Neutral	9.1	14.6	12.9	5.9	12.5	11.1
Agree	63.1	49.1	66.9	78.4	67.9	68.6
Strongly agree	12.9	7.7	6.3	9.8	7.7	9.4
Mean	3.71	3.33	3.63	3.92	3.69	3.75

Regarding attachment to the native place, the proportion of positive emotions (sum the percentages of “strongly agree” and “agree” as the percentage of positive emotions) reported by the participants were: affiliation 85.7%, belonging 76.3%, responsibility 77.4%, concern 88.9%, security 78%, and pride 77%. Overall, emotional attachment to the native place was widespread and scored quite high. Among the six items, the participants rated “concern” (4.03) as the highest and “responsibility” (3.72) as the lowest.

Regarding attachment to the destination place, the proportion of positive emotions reported by the participants were: affiliation 76%, belonging 56.8%, responsibility 73.2%, concern 88.2%, security 75.6%, and pride 78%. Among the six items, the participants rated “concern” (3.92) as the highest and “belonging” (3.33) as the lowest. Migrants and returnees also reported positive emotions toward the destination place, but it is apparent that the magnitude of positive emotions toward the destination place is smaller than that toward the native place.

One notable finding is that the largest gap in ratings among participants was for the “belonging” item (76.3% positive response for native place compared to 56.8% for destination place), even though they also reported positive attachment to their destination place. This result suggests, to some extent, the complex situations encountered by rural-to-urban migrants in adapting to the city environment. Cities provide opportunities for leisure, entertainment, learning, and self-realization for rural migrants, and when the destination provides functional support for individual pursuits or specific activities, individuals will value the place, and their level of personal achievement will be reflected in their attachment level (Scannell and Gifford, 2010). This may also explain why “pride” in the destination was slightly higher than for the native place. However, rural migrants who come to the city are not rooted in the local history, geography, and language (Tuan, 2007) and are less likely to develop a sense of identification in a different environment (Du et al., 2018).

**Figure 3:**
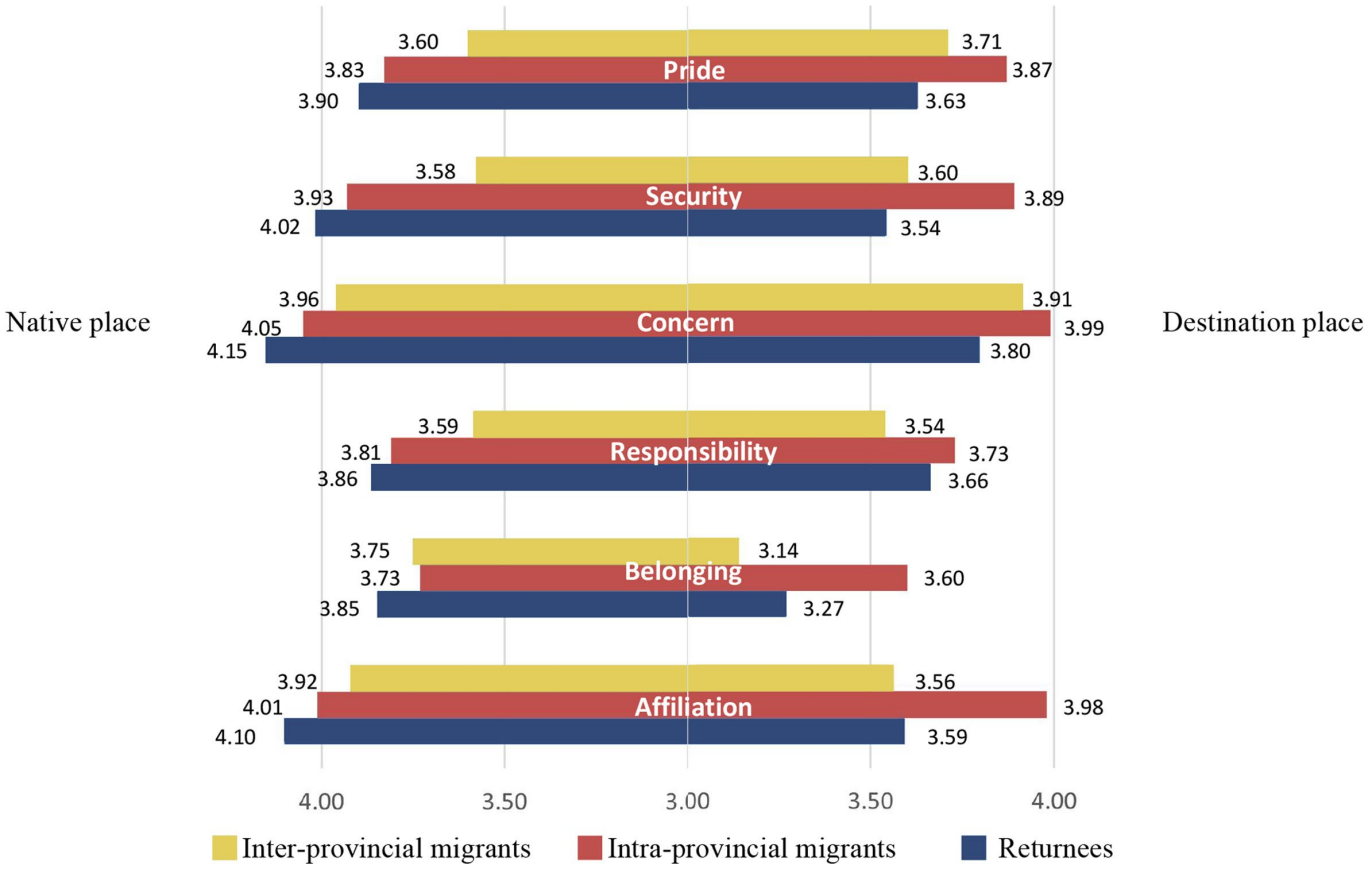
Place attachment among rural migrants and returnees (mean).

When participants were divided into three groups based on their settlement choices (intra-provincial migration, inter-provincial migration, and return) to examine their attachment to native place and destination place, the gradual changes in place attachment intensity were evident (Figure 3). Overall, the attachment to native place and destination place was not markedly different among intra-province migrants, while return migrants showed a greater attachment to native place than destination place. In terms of attachment to native place, the ratings decreased gradually among return migrants, intra-province migrants, and inter-province migrants, with only the belonging item showing higher ratings among inter-province migrants than intra-province migrants. In terms of attachment to destination place, intra-province migrants gave higher average ratings than the other two groups. Only in the items of pride, security, and concern did inter-province migrants give higher ratings than return migrants. Surprisingly, inter-province migrants had higher average ratings than intra-province migrants on both the belonging and affiliation items for native place, which may reflect a strong attachment to their hometown and a difficulty in integrating into a new environment.

### 4.2 Place attachment: factorial structure

**Figure 4:**
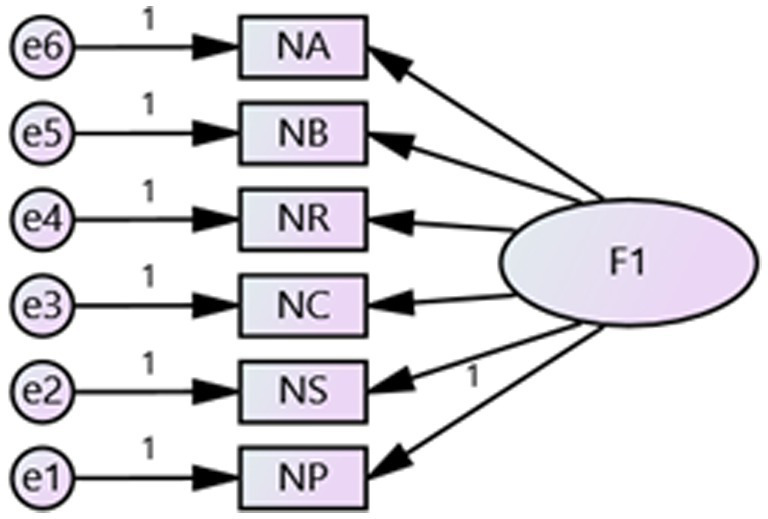
Confirmatory factor analysis: model of native place.

**Figure 5:**
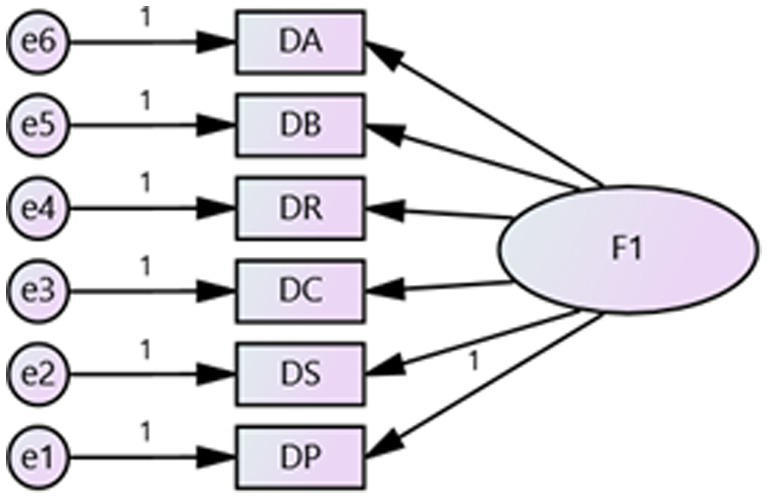
Confirmatory factor analysis: model of destination place.

Cronbach’s alpha was used to test the reliability of the scale (Table 2). High internal consistency was found in both the native place attachment (alpha = 0.813) and destination place attachment (alpha = 0.840) subscales. Removing any item would decrease Cronbach’s alpha, indicating that the scale has a reliable dimension. For both places, a clear two-factor solution accounted for 55.99% of the variance in the scale. Table 2 also shows that all six items loaded positively on place attachment, with acceptable factor loadings ranging from 0.630 to 0.781. The Security item had the strongest association with native place attachment, while Belonging had the strongest association with destination place attachment. The KMO value of 0.841 indicates high collinearity between variables, demonstrating that the data were suitable for factor analysis. Confirmatory factor analysis was used to evaluate validity (see Figures 4, 5), with both places and all 12 items tested using AMOS 24.0. While the RMSEA index is only marginally within the acceptable range, other indicators have passed the tests, showing the model’s excellent fit. Therefore, the validity of the scale has been verified (Table 3).

**Table 2:** The scale of place attachment: factor loading.

	Native place (*N* = 287)			Destination place (*N* = 287)		
	Corrected item-total correlation	Alpha if item deleted	Factor loading	Corrected item-total correlation	Alpha if item deleted	Factor loading
Affiliation	0.501	0.799	0.630	0.674	0.803	0.773
Belonging	0.452	0.811	0.640	0.632	0.815	0.781
Responsibility	0.588	0.781	0.711	0.548	0.828	0.651
Concern	0.642	0.775	0.752	0.586	0.824	0.717
Security	0.643	0.768	0.776	0.670	0.804	0.755
Pride	0.655	0.765	0.752	0.640	0.811	0.755
Reliability coefficients (Cronbach’s Alpha)		0.813			0.840	
Variance explained (%)						55.99%
KMO Measure of Sampling Adequacy						0.841

Extraction Method: Principal Component Analysis. Rotation Method: Varimax with Kaiser Normalization.

**Table 3:** Confirmatory factor analysis: goodness-of-fit indices.

	cmin/df	RMSEA	GFI	CFI	AGFI	NFI	TLI
Destination place	1.306	0.033	0.987	0.995	0.969	0.980	0.992
Native place	2.827	0.800	0.981	0.923	0.935	0.970	0.951

### 4.3 Place attachment: typology

After verifying the reliability and validity, the Place Attachment scale and data were found to be reliable and suitable for factor analysis. Maximum likelihood was used as the extraction method, and Bartlett scores were used to obtain the factor scores for each case on the two locations, reflecting the strength of each case’s attachment to the two locations. Subsequently, a two-step cluster analysis was conducted on the two subsamples (migrants and returnees). Each individual’s factor scores within these five clusters were standardized. Then, by calculating the mean of the standardized factor scores for each cluster, we derived the positive or negative place attachment values, as displayed in Table 4.

**Table 4:** Clusters of migrants and returnees by attachment (standardized mean value).

	Inter-provincial and intra-provincial migrants			Returnees	
	Adapters	Nostalgics	Embracers	Reluctants	Detacheds
Native place	0.34	0.56	−1.39	0.20	−0.55
Destination place	0.36	−1.61	0.32	0.52	−1.39
*N*=	137	41	50	43	16
%	60.1%	18.0%	21.9%	72.9%	27.1%

The subsamples of both provincial and non-provincial migrants were subjected to two-step cluster analysis based on their scores for the two variables of attachment to native place and destination place. SPSS generated three clusters, with Silhouette Measure of Cohesion and Separation ranging from 0.5 to 1 and an average silhouette value of 0.6 indicating good cluster quality. These clusters of migrants were as follows: (1) Adapters: a cluster with high attachment to both native (0.34) and destination places (0.36); (2) Nostalgics: a cluster with high attachment to native place (0.56) and low attachment to destination place (−1.61); and (3) Embracers: a cluster with low attachment to native place (−1.39) and high attachment to destination place (0.32) (Table 4).

For the subsample of returnees, cluster analysis was also performed based on the two variables of attachment to native place and attachment to destination place. SPSS 23.0 generated two clusters, with Silhouette Measure of Cohesion and Separation ranging from 0.5 to 1 and an average silhouette of 0.6, indicating good clustering quality. The two clusters are as follows: (1) Settlers, who exhibit high attachment to both native place (0.20) and destination place (0.52), but with a significantly lower attachment to native place compared to destination place; and (2) Detacheds, who exhibit low attachment to both native place (−0.55) and destination place (−1.39), but with a markedly lower attachment to destination place compared to native place (Table 4).

### 4.4 Attachment and demographic variables

Table 5 presents the demographic characteristics of the five categories of rural migrants in the sample, along with the results of the chi-square tests. For the migrant subsample, which includes both internal and external migrants, there were no significant differences in age, gender, or monthly income across the three subgroups. However, in several items such as whether they own property in their native or destination places, marital status, and having children, all expected counts were greater than 5, and the total sample size *N* = 282>40, indicating significant differences in these items. Regarding property ownership in their native place, Nostalgics had the highest ownership rate while Embracers had the lowest. The chi-square test yielded a value of 14.849a, and the minimum expected count was 17.80 (*p* = 0.001). In terms of property ownership in their destination place, Embracers and Adapters had higher rates than Nostalgics. The chi-square test yielded a value of 34.982a, and the minimum expected count was 9.53 (*p* = 0.000). Regarding the items of marital status and having children, Adapters had higher rates than the other two groups. Embracers had a slightly higher marriage rate than Nostalgics, but a slightly lower rate of having children. Analysis showed that these two items also had significant differences, with a value of 11.133a and *p* = 0.008 for marital status, and a value of 10.867a and *p* = 0.004 for having children. As for the education level item, since four cells (33%) had expected counts less than 5, the minimum expected count was 1.60, and after Fisher’s exact test was conducted, the result was *p* = 0.000. The differences were significant among the leavers subsample, with Nostalgics having a higher rate of high school education or below than Adapters and Embracers, while Embracers and Adapters had higher rates of university education or above than Nostalgics. As for the returnees subsample, the chi-square test results showed no significant differences in sociodemographic characteristics.

**Table 5:** Chi-square test of socio-demographic characteristics by cluster.

	Migrants					Returnees			
	Adapters	Nostalgics	Embracers	Chi-square value	*p* value	Reluctants	Detacheds	Chi-square value	*p* value
Male (%)	43.8	51.2	48.0	0.798	0.671	51.2	31.3	1.863	0.172
Age (%)				12.387	0.135	11.6	25.0	2.347	0.504
18–35	23.4	41.4	38.0			14.0	18.8		
36–50	29.9	19.5	14.0			72.1	56.3		
50~	46.7	39.0	48.0			2.3	0.0		
Education Level (%)				17.384	0.008			1.128	0.569
~ Junior high school	3.6	9.8	0.0			7.0	12.5		
high school	8.8	22.0	4.0			23.3	12.5		
bachelor	75.9	63.4	78.0			69.8	75.0		
Graduate or above	11.7	4.9	18.0			0.0	0.0		
Monthly income				13.495	0.096			1.860	0.761
~2,500	2.9	9.8	0.0			7.0	12.5		
2,500–4,000	10.9	14.6	12.0			34.9	25.0		
4,000–10,000	54.0	43.9	40.0			53.5	50.0		
10,000–20,000	22.6	19.5	26.0			2.3	6.3		
20,000~	9.5	12.2	22.0			2.3	6.3		
Household in NP (%)	46.0	61.0	22.0	14.849	0.001	60.5	62.5	0.020	0.887
Household in DP (%)	83.9	41.5	86.0	34.982	0.000	67.4	56.3	0.637	0.425
Married (%)	92.0	73.2	80.0	11.133	0.004	95.3	81.3	2.988	0.084
Having child (%)	90.5	78.0	72.0	10.867	0.004	95.3	87.5	1.137	0.286
Age of leaving NP (%)				22.509	0.013			1.447	0.919
~10	9.5	4.9	6.0			7.0	6.3		
11–15	2.2	12.2	4.0			4.7	0.0		
16–20	13.1	14.6	20.0			11.6	18.8		
21–25	56.2	31.7	48.0			41.9	37.5		
26–30	10.9	9.8	10.0			14.0	18.8		
30~	8.0	26.8	12.0			20.9	18.8		
Years outside of NP (%)				24.915	0.006			4.423	0.490
~5	5.1	12.2	4.0			32.6	37.5		
6–10	18.2	24.4	32.0			11.6	12.5		
11–15	13.1	29.3	4.0			2.3	0.0		
16–20	7.3	7.3	8.0			4.7	18.8		
21–25	14.6	12.2	8.0			4.7	0.0		
25~	41.6	14.6	44.0			44.2	31.3		

NP, Native place; DP, Destination place.

In terms of other variables, the age at leaving home yielded five cells (27.8%) with expected counts less than 5 in the chi-square test, and the minimum expected count was 1.80. After performing Fisher’s exact test, the result showed a significant difference in the age at leaving home among the migrants (*p* = 0.013). The proportion of Nostalgics who left home before the age of ten was lower than that of Adapters and Embracers, while the proportion of Nostalgics who left home after the age of 30 was significantly higher than that of Adapters and Embracers. For the number of years spent outside their hometown, this variable yielded four cells (22.2%) with expected counts less than 5 in the chi-square test, and the minimum expected count was 2.52. After performing Fisher’s exact test, the result showed a significant difference in the number of years spent outside their hometown among the returnees (*p* = 0.000). The proportion of Nostalgics who spent less than 5 years outside their hometown was higher than that of Adapters and Embracers, while the proportion of Adapters and Embracers who spent more than 25 years outside their hometown was significantly higher than that of Nostalgics. As for the sample of returnees, the chi-square test results showed no significant differences in age of leaving home and years of staying outside.

## 5 Discussion

Existing literature on rural migration in China has primarily focused on the attachment and integration of rural migrants in large cities, neglecting the importance of their native place. This oversight makes it challenging to fully comprehend the attachment experiences of rural migrants and returnees, as well as the compatibility and conflicts between their migration and place attachment. The contribution of this paper to the rural migration and place attachment literature lies in the investigation of rural migrant and returnee samples from the same region while simultaneously testing their place attachment scales for both native and destination places. This approach allows for a more comprehensive understanding of the relationship between migrants and places, and it highlights the potential conflicts and compatibility that migration brings to place attachment.

Both rural migrants and returnees reported positive attachment to their native place and destination place. However, a comparative analysis revealed that the attachment scores for native place items, except for pride, were consistently higher than those for destination place items among migrants and returnees, with the largest difference found in the belonging category. This observation suggests that attachment to native and destination places does indeed vary. Discrimination against rural migrants is well-documented in the literature (Chen, 2013). Although official documents and media have ceased the public use of discriminatory terms toward rural migrants, they are still regarded as second-class citizens in contemporary urban contexts (Du, 2018). Discrimination from urban local residents, combined with a systematic non-acceptance of rural migrants (Hukou), severely hampers their integration into city life. This might result in a stronger sense of belonging to their native place over their destination place for migrants from outside the province. For rural migrants, settling in the city is challenging; it requires significant effort to achieve personal development success, thereby securing urban residence and overcoming their discriminated social status. The sense of achievement tied to success is, to some extent, linked with settling in the city, which could explain why migrants and returnees score higher on pride for their destination place than for their native place. The results highlight the strength of place attachment in rural areas and the challenges rural migrants face in forming attachments to their destination places. These findings align with previous literature that posits stronger attachments to rural communities compared to larger, particularly urban, communities (Lewicka, 2005). At the same time, attachment to a place is not limited to one location; attachment to a destination place can coexist with attachment to a native place. After leaving one place, individuals can develop emotional connections with another or multiple other places, demonstrating the flexibility and compatibility of place attachments.

In terms of attachment to the native place, a progressive decrease is observed among returnees, intra-provincial migrants, and inter-provincial migrants. Conversely, for destination place item scores, apart from responsibility, belonging, and affiliation, a progressive decline is noted in the order of intra-provincial migrants, inter-provincial migrants, and returnees. We believe this reflects the differing place attachment patterns between migrants and returnees. Migrants exhibit a stronger attachment to their destination places than returnees, while returnees display a stronger attachment to their native places than migrants. The intensity of attachment is related to the decision to return or not (Riethmuller et al., 2021). Simultaneously, we can observe that geographical migration does not necessarily destroy place attachment but may weaken its intensity. The attachment preferences of migrants and returnees demonstrates this point. Furthermore, this finding underscores the conflict and compatibility between migration and place attachment, enriching our understanding of their complex relationship.

The study’s findings reveal noteworthy differences in the experiences and emotions of intra-province migrants and inter-province migrants. These disparities can be attributed to several factors, with one of the most prominent being the cultural differences between provinces. It is important to note that cultural distinctions tend to be more pronounced between provinces than among cities within the same province. This heightened inter-provincial cultural diversity can have a profound impact on the migrants’ emotional ties to their hometowns. Inter-province migrants often experience a more significant cultural shift when they move to a new province. This shift can include variations in language, traditions, customs, and even cuisine. As a result, inter-province migrants may find themselves feeling a stronger attachment to their place of origin due to the contrast in culture and the longing for their familiar cultural environment. In contrast, intra-province migrants, while still experiencing some cultural variations, are more likely to encounter similarities in culture and customs compared to inter-province migrants. This may lead to a relatively smoother transition and adaptation to their new destination within the same province. Consequently, the emotional connection of intra-province migrants to their hometowns may not be as pronounced as that of their inter-province counterparts. Additionally, the emotional attachment of inter-province migrants to their hometowns may also be influenced by distance and accessibility. Provinces typically cover larger geographical areas, and the physical distance between their new residence and their hometowns can be more substantial. This distance can intensify the longing for their place of origin and contribute to a deeper emotional connection.

As the results indicate, based on attachment intensities to the native place and destination place, a two-step cluster analysis reveals three types of migrants and two types of returnees. The three types of migrants include Adapters, with medium attachment to both native place and destination place; Nostalgics, with a higher attachment to the native place and a lower attachment to the destination place; and Embracers, with a lower attachment to the native place and a higher attachment to the destination place. The two types of returnees are Settlers, with high attachment to both native place and destination place but a notably lower attachment to the native place, and Detacheds, with low attachment to both native place and destination place but a significantly lower attachment to the destination place. The results show diverse attachment types among rural migrants and returnees. In today’s era of mobility, attachment to specific locations exhibits apparent individual differences. For Adapters and Embracers, who have left the rural areas and developed stronger attachments to new environments, They actively chose the place of attachment. They deliberately choose to live in a preferred place, setting their low or medium attachment to the native place aside. Nostalgics display a Place alienation attachment type, related to their dislike of their current place of residence. Detacheds have not established a positive emotional connection with any place. As for Settlers, they prefer the destination place compared to their current native place, revealing a contradiction and a certain degree of acceptance regarding their place of residence. While we were inspired by the literature that categorizes based on levels of attachment, our survey participants, the scope of places considered, and the criteria we relied on differ from those mentioned in the literature review that also categorizes based on attachment levels. Therefore, a direct comparison with literatures was not made in our discussion. This highlights the innovative aspect of our research. Specifically, Hummon’s survey focused on a single location, whereas ours encompasses two (Hummon, 1992); Lewicka investigated place attachment among transnational migrants in Poland and Europe (Lewicka, 2011a), while our focus is on rural migrants and returnees; Jamieson employed interviews to explore place attachment at a single location (Jamieson, 2000), in contrast, we conducted a quantitative survey across two locations; Engbersen examined national attachment among transnational laborers across European countries (Engbersen et al., 2013), our survey targeted migrants and returnees within rural area; Du investigated urban residents’ migration behavior using place attachment and belonging as variables for clustering (Du, 2017). In summary, our standards for categorization are completely distinct, and the vast differences in the scale of survey participants and study areas preclude any direct comparison between their attachments.

In terms of sociodemographic variables, significant differences exist among migrant subgroups in aspects such as property ownership in both locations, marital status, whether they have children, and educational level. The higher the education level, the lower the proportion of Nostalgics, and the higher the proportion of Embracers. As Tuan mentioned more than 40 years ago, with the increase in education levels, their scope of identity shifts from neighborhoods and nations to regional and global levels (Tuan, 1977). Lewicka also found that for people with an “ideological rootedness” attachment type, place attachment is positively linearly correlated with education (Lewicka, 2013). Regarding property ownership in the native place, Nostalgics significantly outnumber Adapters and Embracers, while, correspondingly, Nostalgics significantly lag behind Adapters and Embracers in property ownership at the destination place. Property ownership has consistently been a sociodemographic factor in predicting place attachment (Bolan, 1997). Marriage and having children variables have not been mentioned in previous literature. Adapters score higher than Nostalgics and Embracers in these two aspects, which may be because, within the narrative of Chinese familism, marriage signifies the merging of two extended families, and raising children strengthens the intergenerational exchanges between families, thereby fostering closer connections among family members. In terms of age of leaving home and years of outside, there are significant differences among migrants in terms of age at leaving home and years spent outside their hometown. Higher age of leaving the native place indicates a longer period of residence in the hometown, and a bigger number of years outside of the native place suggests a longer duration of residence in the destination place. The changes in Nostalgics in these two variables support this notion, as the older the age at first leaving home, the higher the proportion of Nostalgics. The longer they have been away from their hometown, the lower the proportion of Nostalgics. The positive correlation between time spent living in a place and attachment to that place makes it one of the standards for measuring attachment to a place (Kleit and Manzo, 2006).

There are still two Issues to be noted in this study. First is the issue of differentiated migration. Different migration flows play a dominant role in the life cycle of rural environments (Laoire and Stockdale, 2016). This article only considers migration from rural areas to cities, and the rural–urban dichotomy presents a problem, especially since internal population movements between rural areas have been observed in Europe (Bijker et al., 2013). The proportion of this population in Chinese rural migration and the sampling criteria are still unclear, which may need to be addressed in future research on rural migration in China. In addition, migrants may not move only once, and may also develop attachments to more than one destination place. If we intend to investigate the number of migrant moves and attachments to multiple locations, additional research methods may need to be employed. The technological tools that have emerged in the information age can help us to make up for the limitations of this study, and considering the impact of mobile networks, the use of mobile data to follow up on rural migrants is one of the possible ways to do so, and a long-term follow-up with adequate protection of privacy and the consent of the study participants themselves is one of the future directions of the study.

The significance of this study lies in the fact that rural migrants and returnees have experienced living in both rural and urban environments, and have developed emotional connections in both contexts. By considering the needs and psychological differences of returnees, as well as the concerns and practical challenges faced by migrants, policies can be designed to benefit both groups. The government and society can help rural migrants and returnees to better adapt and integrate into their new environments through various measures. These measures could include providing vocational training and employment opportunities, increasing social and cultural exchange opportunities, improving infrastructure in both urban and rural areas, among others. Furthermore, the government and society can encourage returnees to actively participate in local development and construction, leveraging their experience and resource advantages to contribute to the economic and social development of their hometowns. Such measures can enhance the sense of place attachment for both rural migrants and returnees, promote social harmony and stability, and foster sustainable economic development.

## 6 Conclusion

Overall, both migrants and returnees exhibit positive place attachment toward their place of native and destination. Returnees tend to have a stronger place attachment to their hometown compared to migrants, while their place attachment to the destination city is weaker in comparison to migrants. This indicates that the act of migration, to a certain extent, influences an individual’s formation of place attachment, and that the object of attachment is not solely fixed to a particular location. Some migrants and returnees decide to settle in places where they do not feel a sense of place attachment, suggesting that place attachment is not the sole determinant in the decision-making process of migration or settlement. The impact of specific attachments on the migration of rural migrants and returnees could be the subject of a follow-up study. This paper contributes to the research literature on migration and place attachment by distinguishing itself from big-data-style quantitative studies of urban perspectives by taking a place-of-origin perspective, We believe that this paper makes a substantive contribution to the literature on environmental psychology. Based on the results of our study, we recommend that urban administrators should focus on increasing communication and interaction with the rural migrant population, in order to enhance their sense of belonging. In terms of rural policies, the returnee group, with their strong attachment to the countryside, should be leveraged by utilizing their work experience in big cities to create conditions conducive to the development of rural areas. For example, in Shuangfeng County, the study area of this paper, as a county where the proportion of the population living in rural areas has dropped to less than 45%, the primary consideration is to retain the outflow of population and to encourage returning migrants to contribute to their hometowns with the experience they have gained abroad. In the next Spatial Planning and Economic Development Planning, targeted planning measures should be proposed in the “Talent people planning” section. For example, provide tax exemptions for talented people who are willing to return to the countryside to encourage them to return to their hometowns for development. Or in the planning for the return of talents to set up enterprises to provide exclusive construction land for the construction of office space or factories, while providing preferential policies on corporate income tax. In the literature reviewed in this paper, we know that the economic gap between urban and rural areas is an important cause of population outflow, and if this gap can be narrowed through planning policies, more migrants will be attracted to return. At the same time, for the existing population and space, it is possible to consider planning the space and lifestyle of collective housing to establish a rural living community, which will facilitate better services for existing rural residents.

## Data availability statement

The raw data supporting the conclusions of this article will be made available by the authors, without undue reservation.

## Author contributions

LH: Conceptualization, Data curation, Formal analysis, Investigation, Methodology, Project administration, Resources, Software, Validation, Visualization, Writing – original draft, Writing – review & editing. YM: Conceptualization, Formal analysis, Validation, Writing – review & editing. TK: Supervision, Validation, Writing – review & editing.
